# A direct view of the complex multi-pathway folding of telomeric G-quadruplexes

**DOI:** 10.1093/nar/gkw1010

**Published:** 2016-10-30

**Authors:** Mikayel Aznauryan, Siri Søndergaard, Sofie L. Noer, Birgit Schiøtt, Victoria Birkedal

**Affiliations:** 1 Interdisciplinary Nanoscience Center (iNANO), Aarhus University, Gustav Wieds Vej 14, 8000 Aarhus, Denmark; 2 Department of Chemistry, Aarhus University, Langelandsgade 140, 8000 Aarhus, Denmark

## Abstract

G-quadruplexes (G4s) are DNA secondary structures that are capable of forming and function *in vivo*. The propensity of G4s to exhibit extreme polymorphism and complex dynamics is likely to influence their cellular function, yet a clear microscopic picture of their folding process is lacking. Here we employed single-molecule FRET microscopy to obtain a direct view of the folding and underlying conformational dynamics of G4s formed by the human telomeric sequence in potassium containing solutions. Our experiments allowed detecting several folded states that are populated in the course of G4 folding and determining their folding energetics and timescales. Combining the single-molecule data with molecular dynamics simulations enabled obtaining a structural description of the experimentally observed folded states. Our work thus provides a comprehensive thermodynamic and kinetic description of the folding of G4s that proceeds through a complex multi-route pathway, involving several marginally stable conformational states.

## INTRODUCTION

Telomeres are unique nucleoprotein complexes that occur at the termini of eukaryotic chromosomes and are essential for the maintenance of genome integrity and prevention of chromosome degradation and fusion (1). Telomeric DNA is double stranded, consisting of multiple repetitive guanine-rich units (TTAGGG in humans), terminated by ∼300 nucleotide (nt) single-stranded overhang at the 3′-end (1). *In vitro*, short guanine-rich oligonucleotide sequences can fold into non-canonical DNA secondary structures, called G-quadruplexes (G4s) (2). Over the last decade a large number of proteins and enzymes participating in the regulation of G4 formation (and disruption) have been identified both *in vitro* and *in vivo* (3,4). More recently, several groups reported evidences of G4 formation in the genome of eukaryotic cells (5,6). The formation of G4s from exogenous G4 forming DNA sequences was also observed in live *Xenopus laevis* oocytes (7,8). In addition, a novel high-throughput sequencing technology allowed identifying a vast number of G4 structures and mapping their distribution throughout a human genome (9). Taken together, these observations strongly support the occurrence and functional relevance of G4s *in vivo*. G4s have been proposed to play a regulatory role in a number of cellular processes such as telomere maintenance, DNA replication, recombination and gene expression (4). They have been found to be involved in several oncological (10) and neurological (11) diseases and are potential targets for pharmacological intervention, utilizing specific G4-binding ligands (10,11). G4 structures are also used in a number of applications for DNA nanotechnology (12).

Despite the simplicity of the short telomeric G4 forming sequences, G4 structures have an intrinsic conformational polymorphism, due to differing backbone orientations and type of internal loops (13,14). The prevalence of one particular conformation in solution is dictated by factors such as counter-ion type and concentration, solvent composition, as well as the length of the DNA sequence and the type of terminal flanking nucleotides (15). Depending on these factors several stable folded G4 structures have been identified for the human telomeric sequence (16–20). This multifactorial nature of G4s makes their folding highly complex, possibly involving a number of stable intermediate states and misfolded species and is beyond a simple two-state approximation (21). Several studies have found G4 folding to occur through a simple sequential pathway, involving up to two intermediate states (21–26), however recently more complex folding scenarios have started to emerge (27–30). One important bottleneck in uncovering the G4 folding pathway remains the structural identification of the different folded states appearing during the folding process.

To address this problem we have utilized single-molecule Förster Resonance Energy Transfer (FRET) microscopy in combination with molecular dynamics (MD) simulations. Single-molecule FRET microscopy allows real-time monitoring of the folding of single G4 molecules, omitting both time and population averaging (31) and thus provides a direct view of G4 conformational diversity and dynamics. Whereas, combining the single-molecule FRET data with MD simulations allowed obtaining the structural description of the experimentally observed G4 states. We find that the hybrid 1 conformation represents the predominant long-lived folded conformation of the human telomeric sequence in potassium containing solutions. Before reaching this conformation, several alternative off-pathway folding transitions occur into marginally stable anti-parallel chair, 2-tetrad anti-parallel basket and hybrid 2 conformations.

Thus, our work provides a comprehensive understanding of the folding process of the human telomeric G4s *in vitro* that may be essential for elucidating the microscopic details of regulation of G4 structures by G4-binding chaperone proteins and resolving enzymes, interactions of G4s with small-molecule ligands and their overall function.

## MATERIALS AND METHODS

### Sample preparation

All fluorescently labeled DNA oligonucleotides were purchased from IBA (Germany) as a HPLC and PAGE purified product (Supplementary Table S1). The G4 forming oligos include a 22 (or 23) nucleotides overhang that was terminally (3′) labeled with Cy3 via amidite coupling. In case of the hTelo-I14 sequence the last guanine of the overhang was labeled with Cy3 via NHS coupling to the amino-C6 dG. The complementary duplex forming oligo was labeled with Cy5 via NHS coupling to the amino-C6 dT. It also contained a biotin at the 3′-end used for surface immobilization.

The G4 forming and complementary oligos were mixed with a ratio of 1.2:1, respectively, to a final concentration of 5 μM in the annealing buffer (20 mM Tris–HCl buffer (pH 7.5), with 50 mM LiCl or KCl). The sample was thermally annealed in a water bath, by keeping it at 95°C for 5 min, followed by gradual cooling to room temperature over 24 h.

### Single-molecule FRET experiments and data analysis

Single-molecule FRET experiments were performed on surface-immobilized molecules employing a prism-based total internal reflection microscope (Zeiss). Labeled molecules were immobilized inside a coverslide chamber (a pair of quartz and glass slides assembled together by Parafilm stripes) using a BSA-biotin and streptavidin anchoring. Stock solutions of the labeled sample with a concentration of ∼5 pM were used for immobilization. The excess of non-immobilized molecules was washed out by flushing the chamber with dilution buffer (20 mM Tris–HCl buffer (pH 7.5), with 25 mM LiCl or KCl). Detailed protocols of these experimental procedures are published elsewhere (32). Prior to imaging, the coverslide chamber was flushed with an imaging buffer consisting of the dilution buffer supplemented with an oxygen scavenging system composed of 2 mM Trolox (Sigma Aldrich), glucose oxidase (Sigma Aldrich, 17 U/ml), catalase (Sigma Aldrich, 260 U/ml) and glucose (Sigma Aldrich, 4.5 mg/ml). Fresh imaging buffer was flushed into the chamber every 20 min, to avoid pH gradients. The excitation of fluorophores was achieved using an alternating laser excitation scheme (33) with 514 nm Ar-ion and 630 nm dye lasers. Fluorescence from the donor and acceptor fluorophores was spatially separated onto the EMCCD camera (Andor, iXON 3) by a wedge mirror. Movies were recorded with a 200 ms integration time per frame with a total length of 600 s (10 min) and were analyzed using the home-built iSMS software (34). Only the trajectories with single-step donor and/or acceptor photobleaching, or showing clear anti-correlated donor–acceptor dynamics, were selected for further analysis. The FRET efficiencies were obtained from the donor and acceptor fluorescence intensities as:

\begin{equation*}E = \frac{{{F_A}}}{{{F_A} + \gamma {F_D}}}\end{equation*}

where *F_D_* and *F_A_* denote donor and acceptor fluorescence intensities after donor excitation, respectively, that were corrected for background signal, donor leakage (*D* = 0.13) and acceptor direct excitation (*A* = 0.05) contributions. The γ-factor (*γ* = 1.2) was used to account for differences in brightness and detection efficiency for the donor and acceptor fluorophores. The correction factors were determined for all samples and a single average value of those was used for correcting the transfer efficiencies for all datasets.

**Figure 1.**
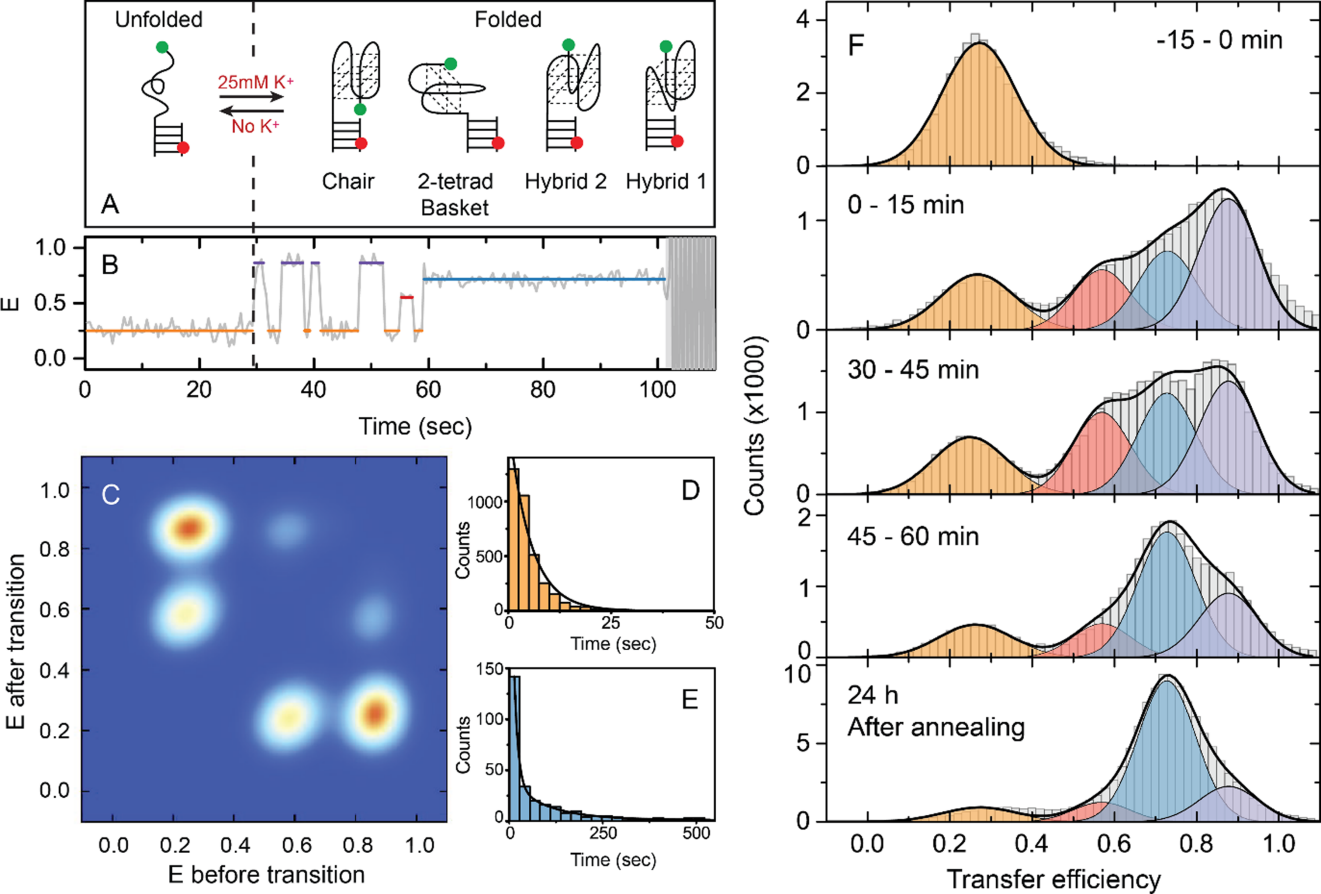
Cation-induced folding dynamics of the human telomeric G4 in 25 mM KCl. (**A**) Schematic representation of the folding of hTelo into various G4 conformations. Green and red circles placed at the G4 structures indicate the Cy3 and Cy5 fluorophores, respectively. (**B**) Representative FRET trajectory of single G4 molecule revealing several E states observed upon G4 folding. Colored solid lines indicate the FRET efficiency of these states as obtained from the HMM analysis. The black dashed line indicates the time of K^+^ addition. (**C**) Transition density plot showing the probability of transitions between different E states. (**D** and **E**) Dwell time histogram of the E≈0.3 and E≈0.73 states fitted with a single- and double-exponential functions, respectively (black lines). (**F**) Representative transfer efficiency histograms of G4 prior addition and for different time periods after addition of 25 mM K^+^, showing the slow kinetics of G4 folding and distribution of several E states. The last histogram was obtained for a sample that was thermally annealed and equilibrated for 24 h. Histograms were fitted globally to a mix of four Gaussian peak functions with shared peak positions and widths for each state (black lines). The color code used for identification of the different states and underlying FRET distributions is the same as in Figure 1B.

Single-molecule FRET histograms were built based on the data arising from molecules containing both active donor and acceptor fluorophores (>100 molecules, except for the histograms shown in Figure 1F corresponding to the 15–60 min times, which are based on 50–60 molecules). All frames of each FRET trajectory (prior the first fluorophore bleaching event) were used to make single-molecule FRET histograms, where each frame yields a count in the histograms.

Single-molecule FRET trajectories showing conformational dynamics were analyzed using hidden Markov modeling (HMM) with the variational Bayesian expectation maximization technique (Figure 1B and Supplementary Figure S1) (34,35). The dwell times corresponding to each G4 conformational state were selected based on the plot of dwell times against transfer efficiency (Supplementary Figure S2). To avoid artefacts of the HMM analysis only the states with dwell times longer than two frames were included in further analysis.

For the kinetic analysis and estimation of folding/unfolding rate constants, the individual transitions were grouped based on the states before and after the transition. It should be noted that in this analysis the states that were cut by blinking, photobleaching or terminated by the end of the experiment were not included. The probability of transitions between different states is reported in Supplementary Table S2. Dwell time histograms were then built for each group and were fitted with single-exponential function to extract the rate constants for each transition (Supplementary Figure S3). The obtained rate constants are summarized in Supplementary Table S3. Detailed procedures for thermodynamic analysis are provided in the Supplementary Data and Table S4.

### Molecular dynamics (MD) simulations

MD simulations were performed for the G4-duplex DNA constructs containing the wild-type and mutant chair (PDB ID: 2KM3 and see Supplementary Data for details), 2-tetrad basket (PDB ID: 2KF8), hybrid 1 (PDB ID: 2JSM) and hybrid 2 G4 conformations (PDB ID: 2JSL). All MD simulations were performed with generalized born implicit solvent (GBIS) in NAMD (36) version 2.9 with bonds to hydrogen atoms held rigid using an integration step of 2 fs. The simulation temperature of 298 K was maintained by the Langevin piston method with a damping coefficient of 1 ps^−1^. For the non-bonded interactions a switching function was used with a switching distance of 15 Å and with an 18 Å cutoff. The AMBER parm99bsc0 force field parameters with χOL4 corrections (37,38) in CHARMM format have been applied to DNA in all simulations. The DNA terminals were terminated according to standard AMBER topology. The solvent was described by the GBIS algorithm with a dielectric constant of 78.5 and implicit ions were included to a concentration of 0.15 M. In total 1.5–2.0 μs simulation was produced for each of G4 conformations. Sufficient sampling was achieved for all constructs (Supplementary Figure S4). Further details on MD simulations are provided in the Supplementary Data.

### Cluster analysis

For the cluster analysis the G-quartet nucleobases were chosen for alignment. A cutoff of 8 Å for 10 clusters was used with the RMSD function in the VMD clustering plugin by L. Gracia (http://physiology.med.cornell.edu/faculty/hweinstein/vmdplugins/clustering). The results of the cluster analyses are shown in Supplementary Table S5.

### Available volume (AV) and simulated FRET efficiencies

The available volume (AV) approach (39,40) was used to quantify the transfer efficiencies for each construct. For the Cy3/Cy5 FRET pair used here the parameters were assessed by measuring the dye and linker dimensions in VMD and these are shown in Supplementary Table S6. The distances between AV determined fluorophore mean positions were found for all frames. Transfer efficiencies were calculated based on the fluorophore distances using four approaches with different frames of the trajectories as input. Supplementary Figure S5 shows the average distance and average efficiency for all four approaches.

## RESULTS

### K^+^-induced folding of G4

For single-molecule FRET experiments we used a DNA construct consisting of a duplex DNA terminated by a G4 forming overhang AGGG(TTAGGG)_3_, corresponding to the human telomeric sequence (hTelo) that mimics the natural telomeric DNA. The DNA construct was labeled with Cy3 (donor) and Cy5 (acceptor) fluorophores for FRET detection (Figure 1A and Supplementary Table S1). Cation-induced folding of G4s and the subsequent conformational dynamics was monitored upon rapid addition of K^+^ (20 mM Tris–HCl and 25 mM KCl, pH 7.5), while imaging on the microscope (Figure 1B). The sample was prepared and initially imaged under non-folding conditions (20 mM Tris–HCl and 25 mM LiCl, pH 7.5) and an unfolded state with low transfer efficiency (E) state (E≈0.3) was initially populated. Upon addition of K^+^, we observe a rapid increase of transfer efficiency as a result of G4 formation (Figure 1B). Thereafter, the majority of FRET trajectories show multiple transitions between several E states, reflecting the highly dynamic nature of G4 folding (Figure 1B and C). Four distinct E states were identified through an HMM analysis of the FRET trajectories (Figure 1B and Supplementary Figure S1). The lowest E state (E≈0.3) represents the unfolded overhang and the three higher E states centered at E≈0.57, E≈0.73 and E≈0.88 correspond to different folded G4 conformations. These states are in dynamic interconversion with dwell times <10 s, except for the E≈0.73 state, which shows an additional long-lived subpopulation with a dwell time an order of magnitude larger (Figure 1D, E and Supplementary Figure S2). This long-lived state is also evident in FRET trajectories, where after initial conformational dynamics between several transient E states, G4s get stabilized in the long-lived E≈0.73 state (Figure 1B and Supplementary Figure S1). The long dwell time is also evidenced by an increased occurrence of static trajectories with E≈0.73 at later stages after folding initiation (Supplementary Figure S1). In addition to fast conformational dynamics, our single-molecule experiments allowed monitoring the G4 folding kinetics occurring on slower timescales (Figure 1F). Prior to the addition of K^+^, the G4 overhang is unfolded and appears as a single peak at low transfer efficiencies. After addition of K^+^ three high E peaks become apparent in the histograms. Interestingly, the distribution of these states varies notably within the first 60 minutes after folding initiation. At the early stages the E≈0.88 state is dominant, while after 60 min the E≈0.73 state becomes the major population. The FRET histogram obtained 60 minutes after KCl addition looks very similar to the one obtained in conditions where the population distribution has reached a complete equilibrium (Figure 1F, lowest panel). These results indicate that G4s reach thermodynamic equilibrium rather slowly, adopting the most stable (lowest energy) conformation within several hours after folding initiation in the presence of 25 mM KCl (Figure 1F).

### Structural assignment of the folded states

But what are the actual conformations of the folded G4 states observed during the folding process? To address this question we used several variants of the hTelo sequence, with specific sequence modifications, that are less polymorphic and can potentially allow structural assignment of the observed E states.

**Figure 2.**
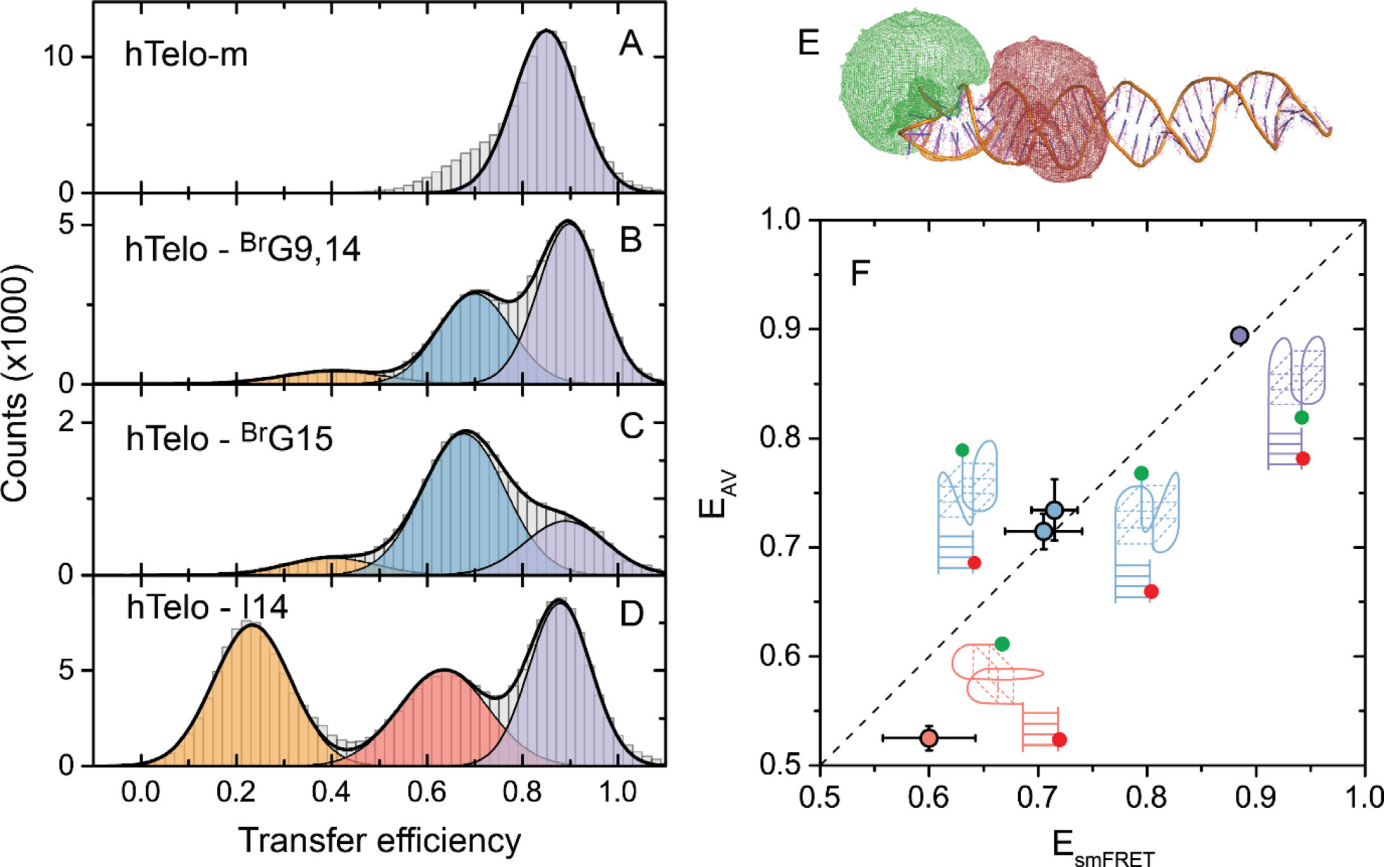
Single-molecule transfer efficiency histograms of G4s formed from hTelo-m (**A**), hTelo - ^Br^G9,14 (**B**), hTelo - ^Br^G15 (**C**) and hTelo-I14 (**D**) sequences in 25 mM KCl. Measurements were performed on samples that were thermally annealed (A–C) or equilibrated for several hours (D). Histograms were fitted to one or a mix of three Gaussian functions (black lines). The color code used for identification of FRET peaks corresponding to different G4 conformations is the same as in Figure 1F. (**E**) Structure of one of the experimental DNA constructs with the hybrid 2 G4 structure (PDB ID: 2JSL) as obtained from molecular dynamics simulations. The positions of the donor and acceptor dyes were estimated using the available volume (AV) approach and are shown as green and red clouds, respectively. (**F**) Transfer efficiencies obtained from single-molecule FRET experiments (E_smFRET_) and AV calculations based on the MD simulations (E_AV_). For each particular G4 conformation (see schematic structures) the E_smFRET_ values represent an average of mean transfer efficiencies obtained for hTelo and corresponding modified sequences. The E_AV_ values are the average of transfer efficiencies for each G4 conformation estimated from four approaches of choosing input frames for the AV calculations (see Supplementary Figure S5 and ‘Materials and Methods’ section for details). The diagonal dashed line shows the identity line.

First, we used a mutant telomeric sequence (hTelo-m) (AGGG(CTAGGG)_3_), which primarily forms an anti-parallel chair conformation in the presence of KCl (41). As expected, one major population is observed in the FRET histogram (Figure 2A). The peak is centered at E≈0.85, which is characteristic for the chair conformation, as previously found in similar buffer conditions (30). The highest FRET state (E≈0.88) of hTelo has a very similar FRET value, strongly indicating that this E state corresponds to the chair conformation.

Next, we used variants of the hTelo sequence, where some guanines were substituted by 8-bromoguanine (^Br^G) derivatives, which preferentially adopt the *syn* orientation (42), allowing to achieve a stabilization of specific G4 conformations (18,43,44). The hybrid 2 structure is expected to be favored for a sequence containing two ^Br^G substitutions at positions 9 and 14 (hTelo - ^Br^G9,14) (44). Three E states were observed in the FRET histogram obtained for this sample (Figure 2B): a minor low E state representing an unfolded G4 and two high E states centered at E≈0.70 and E≈0.88, both corresponding to folded G4s. Taking into account the previous assignment of the E≈0.88 state to the chair conformation, the E≈0.70 state can be assigned to the hybrid 2 conformation. The hybrid 1 conformation is expected to be favored for the hTelo - ^Br^G15 sequence (18). Three FRET peaks were also observed for this sample, with similar transfer efficiencies as for hTelo - ^Br^G9,14 (Figure 2C). The highest FRET state is again attributed to the chair conformation and the dominant peak at E≈0.70 to the hybrid 1 conformation. The hybrid 1 and hybrid 2 conformations are structurally analogous (45) and would likely yield very similar FRET efficiencies, as observed here (Figure 2B and C). Thus, we conclude that the E≈0.73 state observed for the hTelo sequence is represented by a mixture of hybrid 1 and hybrid 2 conformations (Figure 1E). The hybrid 1 conformation is expected to be largely favored for the hTelo sequence (45). We therefore expect that the major long-lived subpopulation (E≈0.73) is likely to represent the hybrid 1 conformation, whereas the short-lived subpopulation can be attributed to the hybrid 2 conformation. Recent time-resolved NMR experiments suggest similar structural assignments for the G4s formed from an analogous telomeric sequence (29). It should be noted that the parallel G4 conformation appears at similar transfer efficiencies (E≈0.71) as the hybrid conformations (Supplementary Figure S6). Therefore, potentially it can also contribute to the E≈0.73 state of the hTelo sequence, however, the formation of a parallel conformation has been observed only in a crystal state or under dehydrating conditions and is unlikely to be observed under our experimental conditions (46,47).

The anti-parallel basket type G4 conformation containing only two tetrads has been previously observed to form from the human telomeric sequence (7). To check if this structure could contribute to the E≈0.57 state, we used a modified telomeric sequence where a guanine at position 14 was replaced by an inosine (hTelo-I14), which has been shown to favor the formation of the 2-tetrad anti-parallel basket conformation (48). Three E states were observed in the FRET histogram obtained for this sample (Figure 2D), where the low and high E states correspond to unfolded and chair conformations, respectively, whereas the intermediate peak appearing at E≈0.6 is expected to originate from the 2-tetrad basket conformation. The E≈0.57 state observed for the wild-type sequence (Figure 1F) is thus assigned to this conformation.

As illustrated by the results above, through careful tailoring of G4-forming sequences we achieve a dramatic reduction of the intrinsic conformational heterogeneity of the wild-type telomeric G4s and a selective favoring of specific G4 conformations that are otherwise only marginally stable. Our single-molecule FRET experiments utilizing these sequences allowed obtaining characteristic signatures of different G4 conformations and thus permitted linking the experimentally observed E states to distinct folded G4 conformations.

### MD simulations

To further aid in the assignment of the observed E states, we performed MD simulations of the DNA construct used in the single-molecule experiments. DNA constructs containing hybrid 1, hybrid 2, 2-tetrad basket and chair (wild-type and mutant) G4s were simulated (Figure 2E and ‘Materials and Methods’ section for details). We find that the G4 structures are predominantly positioned on top of the duplex, i.e. with the G-tetrad plane perpendicular to the long axis of the duplex, for the DNA constructs containing the hybrid 1, hybrid 2 and chair (wild-type and mutant) conformations. However, the 2-tetrad basket conformation is predominantly tilted relative to the duplex, resulting in positioning of the G-tetrad plane almost parallel to the long axis of the duplex (Supplementary Figure S7). This feature is likely to originate due to the structural specificity of the 2-tetrad basket conformation that contains a long diagonal loop that may create some steric restrictions for the G4-duplex relative positioning. To assess the transfer efficiency for each simulated G4 construct we used the AV approach (39) (Figure 2E, Supplementary Figure S5 and ‘Materials and Methods’ section for details). The transfer efficiencies derived from the simulations can be directly compared to those obtained from single-molecule FRET experiments (Figure 2F). The experimental and simulated transfer efficiencies for the chair, hybrid 1 and hybrid 2 conformations match within uncertainty. We even observe a small difference in the simulated transfer efficiencies for the mutant (*E* = 0.84) and wild-type (*E* = 0.89) chair conformations (Supplementary Figure S5), that is also detected in the single-molecule experiments (Figure 2). This reflects the small structural differences of the two constructs and demonstrates the robustness of both approaches of assessing the transfer efficiencies. Due to the specific positioning of the 2-tetrad basket conformation relative to the duplex, the separation between the two fluorophores is larger than for other G4 conformations. It appears in a range very close to the Förster radius (*R*_0_ = 5.6 Å) (Supplementary Figure S5), where the energy transfer efficiency is mostly sensitive to distance changes. This observation can potentially explain the small discrepancy between the experimental and simulated transfer efficiencies for this particular conformation. Overall, we find a remarkable agreement between the two sets of transfer efficiencies obtained from single-molecule FRET experiments and simulations, which further supports our structural assignment.

### Energy landscape for G4 folding

To obtain further mechanistic insight into the folding of G4s we extracted the probability and rate constants for each individual transition observed in the course of G4 folding (Figure 1C, Supplementary Figure S3, Supplementary Tables S2 and 3). We find that the majority of transitions occur between an unfolded and the different folded conformations i.e. the interconversion between folded conformations proceeds via an unfolding step (Figure 1C). Direct transitions between folded conformations were also observed, but are significantly less probable (< 5%) (Supplementary Table S2). It should be noted that direct interconversion between folded G4s is structurally implausible and will require at least partial unfolding (14). Therefore, we believe that the small number of direct transitions observed in our experiments may involve a rapid complete or partial unfolding step that, however, remains hidden due to limited time resolution. We find that the chair, 2-tetrad basket and hybrid 2 conformations are rapidly formed short-lived folded states (*k*_F_ = 0.19-0.21 s^−1^ and *k*_U_ = 0.15-0.35 s^−1^) (Supplementary Table S3) that are extensively observed at the early stages of G4 folding (Figure 1F).

Interestingly, our cation-induced folding experiments reveal that the chair (E≈0.88) is the preferred first populated short-lived folded conformation (80% of all trajectories showing real-time folding) (Figure 1B and Supplementary Figure S1A–D), an observation that is in excellent agreement with results from recent ensemble stop-flow experiments (21). In contrast to more complex structural arrangements necessary for the formation of other conformations, the folding into the chair conformation *per se* can occur through a simple bending of a G-hairpin (transiently populating the unfolded ensemble) (21,22), which may preferentially favor this conformational transition. In the remaining 20% of cases the 2-tetrad basket conformation forms first, which can be explained by the fact that for the formation of this conformation only one K^+^ cation is required. It should be noted, however, that we estimate similar energy barriers for all folding transitions (Supplementary Table S4). In addition, we find that the unfolding of the chair G4 is slower by approximately a factor of two (*k*_U_ = 0.15 s^−1^) compared to the unfolding of the 2-tetrad basket and hybrid conformations. This observation implies kinetic trapping of the anti-parallel chair conformation (49) and can explain the prevalence of this conformation at the beginning of the folding process (Figure 1F).

**Figure 3.**
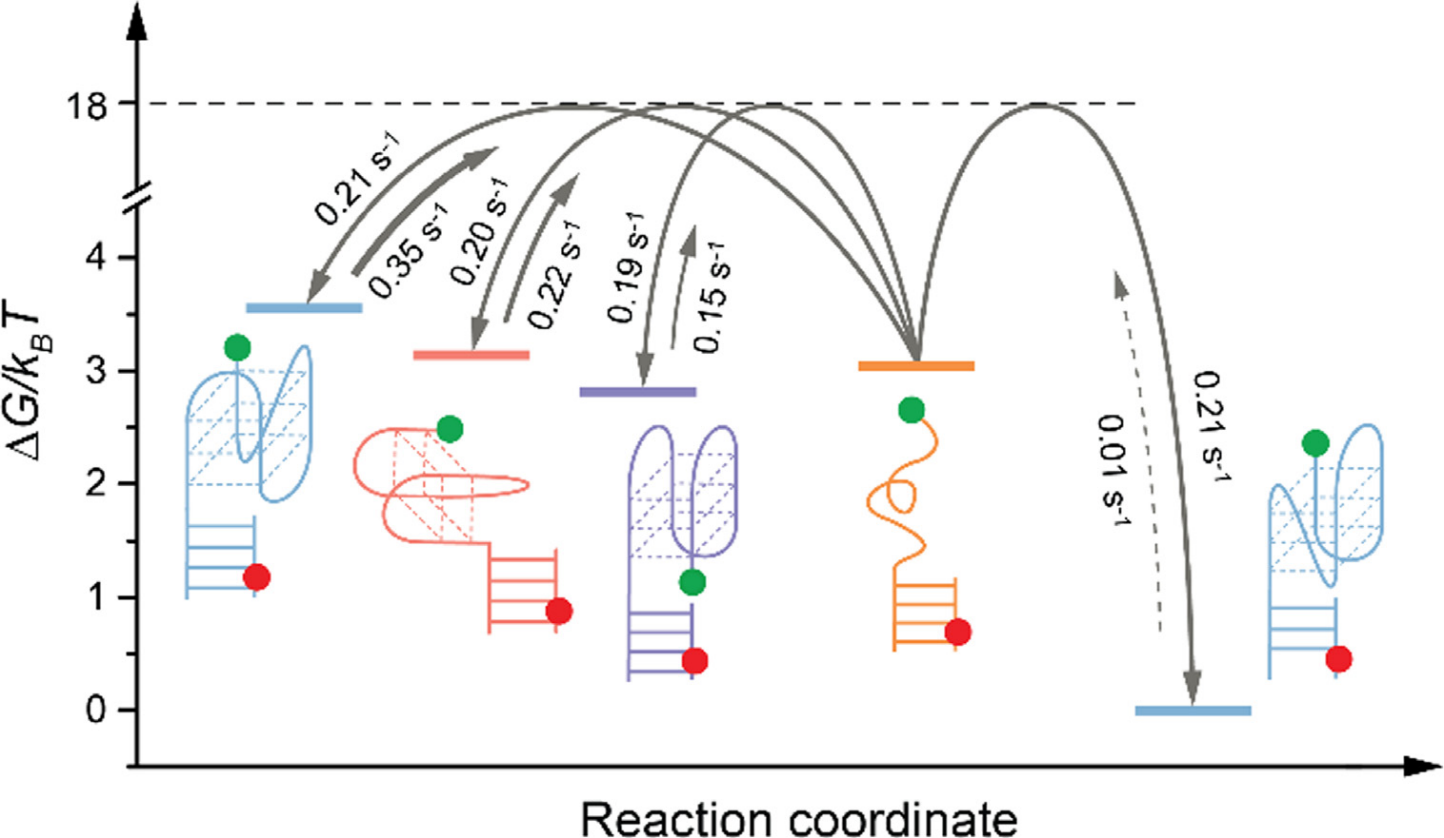
One-dimensional representation of the free energy landscape describing the multi-pathway folding of human telomeric G4s. Horizontal solid lines show energy levels of an unfolded and different folded conformations and the dashed line indicates the estimated height of the energy barrier (see ‘Materials and Methods’ section). Transitions between unfolded and folded conformations are shown as arrows. The thickness of the arrows scales with the value of rate constants of each transition. The slow unfolding transition of the hybrid 1 is shown as a dashed line. Possible transitions between folded conformations are omitted here to avoid overcrowding the figure. The color code of different G4 conformations is the same as in Figure 1B.

Based on the folding/unfolding rates, we can also assess the energetics of the individual conformational transitions observed here (Figure 3 and Supplementary Table S4). The folding transitions to the 2-tetrad basket and hybrid 2 conformations are unfavorable under the current experimental conditions, while the chair is slightly favored. Our results reveal that the hybrid 1 conformation is the most stable and dominant conformation at the folding equilibrium, which stability $(\Delta{G_U}\sim 3\,{k_B}T)$ is in reasonable agreement with the previously reported values from ensemble (50,51) and single-molecule (25) experiments under similar buffer conditions.

## DISCUSSION

By following the entire folding process (hours) of telomeric G4 at the single-molecule level and with a time resolution below 0.5 s, we could resolve several G4 states along the G4 folding pathway and provide a detailed description of their folding energetics and timescales. The combination of single-molecule experiments with different G4 forming DNA sequences and computational modeling of the experimental DNA constructs made possible the identification of several distinct folded G4 conformations of the human telomeric sequence that were not previously simultaneously observed. Single-molecule FRET is a powerful method for resolving several conformational states of complex biological systems that has been used for disentangling the structural polymorphism of telomeric G4s (14,49,52–54). However, previously, only two folded G4 states have been identified through single-molecule FRET experiments, which have been conditionally assigned to parallel and anti-parallel G4 conformations (14,49,52–54). In contrast, we identify FRET signatures of four distinct G4 conformations. The ability to directly resolve such large number of G4 conformations by FRET is not straightforward, due to similar size (*R*_g_∼12 Å) (55) and comparable end-to-end distances for all G4 conformations. The resolution of different G4 conformations is achieved here through a careful design of the experimental system and choice of the specific positions for fluorophore labeling of the DNA constructs. Thus, our integrated approach, utilizing the power of single-molecule FRET microscopy and MD simulations, revealed the formation of two hybrid and two anti-parallel conformations in potassium containing solutions. One of the hybrid (hybrid 2) conformations and both anti-parallel conformations are kinetically favored during G4 folding, whereas the other hybrid conformation (hybrid 1) is the most thermodynamically stable structure for wild-type human telomeric G4s.

In contrast to the moderate thermodynamic stability of G4 structures $(\Delta{G_U} < 3\,{k_B}T)$ the folding energy barriers are relatively large $(\Delta G_F^\ddagger \sim 15\,{k_B}T)$, resulting into a slow folding process (several hours were necessary to reach thermodynamic equilibrium in our experimental conditions). Such high folding energy cost can be explained by an extreme flexibility and large degree of freedom of G4-forming sequences. Indeed, the formation of a planar G-quartet, necessary building block for G4s, may require large structural arrangements of the unfolded single-stranded DNA chain to bring together four guanine bases separated by as many as 20 nucleotides. Interestingly, we find similar folding barriers for all G4 conformations populating the energy landscape. This observation may indicate that the transition state separating the unfolded and folded states is likely to be very similar for all G4 conformations observed here. Although our experiments do not provide any direct structural features of the G4 transition state, we, however, may envision it as an electrostatically stabilized compact structure with similar global fold as for the folded G4s, but lacking most of the native contacts.

We also observe high kinetic barriers for G4 unfolding $(\Delta G_U^\ddagger \sim 15 - 18\,{k_B}T)$ that allow kinetic trapping of folded G4 structures. These thermodynamic features may explain the pronounced structural polymorphism of the G4s, as a result of kinetic partitioning, involving several marginally stable folded G4 conformations.

The observation of direct folded-to-unfolded transitions implies an extremely high degree of cooperativity of G4 folding/unfolding. Such level of cooperativity may be surprising, as previously several partially folded structures, such as G-hairpins and G-triplexes have been proposed to bridge the completely unfolded and folded G4 states, resulting in sequential folding pathways (21–24,26). These structures were also proposed to form during helicase-induced sequential unwinding of G4s (56,57). In our experiments with a G-triplex forming sequence such stable partially folded structures were not detected and are thus unlikely to contribute to any of the high E states here (Supplementary Figure S8). It should, however, be noted that we expect the unfolded state of G4s to be represented by a dynamic ensemble of a number of different unfolded and transiently formed partially folded conformations (29), possibly including G-hairpins and G-triplexes that may appear as a relatively broad peak at low transfer efficiencies in our single-molecule FRET histograms.

Our experimental data obtained at the single-molecule level provides a detailed description of G4 folding as a complex multi-pathway process that involves numerous parallel folding/unfolding transitions and an extensive redistribution between several conformational states. Different site-specific DNA modifications can affect the energetics of the folded states and modify the conformational landscape (Figure 2). Our data show that these modified sequences exhibit similar complex folding behavior, as the one described for the wild-type sequence. The environment and specificity of folding conditions can also influence the G4 folding process. At higher potassium concentrations (100 mM KCl) similar folding behavior and conformational heterogeneity of G4s is observed (Supplementary Figure S9). Under these conditions, the folding of G4s is accelerated, compared to low salt conditions, as well as the marginally stable chair, 2-tetrad and hybrid 2 conformations get more destabilized. Overall, high potassium concentrations favor the formation of thermodynamically stable hybrid 1 conformation and result in reduction of the observed dynamics (Supplementary Figure S9). Studies in sodium-containing solutions show that G4 folding can be very dynamic and follows a multi-pathway process also in these conditions (30,58). These independent observations obtained for different DNA sequences or solutions conditions may point towards common mechanistic features of G4 folding, as described in this work.

An extreme folding dynamics of G4s, as observed here, is certain to impact and possibly modulate their interaction with proteins (59,60) and ligands (61). Although the majority of observed folded states are only marginally stable in current conditions, we expect that they can be individually recognized and trapped through selective structure-specific interactions with different ligands (62) and proteins (63). The structural knowledge available through our experiments can potentially be used to control the folding pathway of G4 structures, i.e. through stabilization of particular G4 conformations via specifically tailored G4-ligand interactions. Therefore, this extreme folding dynamics of G4s should be considered for the development of novel G4-targeting anti-cancer therapies and for elucidating G4-proteins interactions. Our work thus provides a comprehensive thermodynamic and kinetic description of the folding of telomeric G4s, the implications of which may contribute to the structural and energetics framework necessary for future studies of G4 folding *in vivo*.

## Supplementary Material

SUPPLEMENTARY DATA
