# Supplementary material for: A direct view of the complex multi-pathway folding of telomeric G-quadruplexes
Source: Nucleic Acids Res. 2016 Oct 30;44(22):11024–32. doi: 10.1093/nar/gkw1010 (PMC5159523; doi:10.1093/nar/gkw1010)
Supplement: SUPPLEMENTARY DATA [file supp_44_22_11024__index.html]

A direct view of the complex multi-pathway folding of telomeric G-quadruplexes — SUPPLEMENTARY DATA 

# A direct view of the complex multi-pathway folding of telomeric G-quadruplexes

## SUPPLEMENTARY DATA

- SUPPLEMENTARY DATA
