# Supplementary material for: A direct view of the complex multi-pathway folding of telomeric G-quadruplexes
Source: Nucleic Acids Res. 2016 Oct 30;44(22):11024–32. doi: 10.1093/nar/gkw1010 (PMC5159523; doi:10.1093/nar/gkw1010)
Supplement: SUPPLEMENTARY DATA [file supp_gkw1010_nar-02604-f-2016-File005.pdf]

Supplementary Information

A direct view of the complex multi-pathway folding of telomeric G-quadruplexes

Mikayel Aznauryan[a,b], Siri Søndergaard[a,b], Sofie Louise Noer[a], Birgit Schiøtt[a,b] and Victoria Birkedal*[a,b]

[a] Interdisciplinary Nanoscience Center (iNANO), Aarhus University, Gustav Wieds Vej 14, 8000 Aarhus, Denmark

[b] Department of Chemistry, Aarhus University, Langelandsgade 140, 8000 Aarhus Denmark

E-mail: vicb@inano.au.dk

Additional procedures used for performing MD simulations

For MD simulations the G4 structures were taken from the PDB data base and the double stranded DNA duplex was built in Maestro (1). As the PDB structure for the wild-type chair conformation is not available, this structure was built using the hybrid 2 conformation as a template. The difference between the chair and hybrid 2 structures is the direction of the fourth strand stretch (G20-G22) and the loop connecting the third strand stretch (G14-G16) to the fourth (2). The first strand (G2-G4) including the loop (T5, T6, A7) of hybrid 2 was copied and aligned on the third strand to obtain the correct type of connecting loop. After this, G10 was copied and the base of the nucleotide was aligned on the nucleobases of G20 and then G21. G9 was aligned in the same way on G22. This way the correct syn/anti-orientation of each of the nucleotides in the fourth strand stretch was obtained. The resulting model of the wild-type anti-parallel chair was minimized, using a conjugate gradient method, for 10,000 steps before attaching it to the duplex DNA.

The G4 structures were linked to the duplex, such that the G-quartet planes were approximately perpendicular to the long axis of the duplex, and minimized for 10,000 steps. Two K^+ ions were manually placed in the G4 channels. The systems contained ~2,800 atoms each. Since no information describing the rotational state and tilt of the G4s relative to the duplex was found, five rotational states of each G4 attached to the duplex were generated. To produce the rotational G4 states the bond in the junction between the G4 and the duplex (3´ end of last nucleotide in duplex to 5´ end of first nucleotide in G4), which was most parallel to the long axis of the duplex, was selected. The entire G4, including the channel ions, was then rotated around this bond in multiples of 72° resulting in five different conformations. These were all minimized for 10,000 steps. In the few cases where minor overlaps occurred between the G4 and the duplex, nucleotides of the non-minimized rotational state were manually moved slightly and subjected to another minimization of 10,000 steps.

Both restrained and non-restrained MD simulations were performed. In the restrained simulations, a harmonic potential was applied to the distance between the second last base pair in both ends of the duplex to reduce fraying. Fraying occurs naturally in DNA but the lack of water molecules and the destabilizing effect of G4s on double stranded DNA (3) motivated the use of restraints to prevent excessive fraying. The systems were equilibrated for 0.1 ns with a time step of 1 fs and flexible bonds to hydrogens. Restraints on the duplex were applied. For each of the five rotational states the restrained and non-restrained simulations were started from the same equilibration and both run for 150 ns. In total 1.5 µs simulation was thus produced for each type of the four G4 conformations studied. For the mutant anti-parallel chair G4 conformation both the restrained and non-restrained simulations were extended to 200 ns yielding a total of 2.0 µs simulation, however, this did not seem to significantly improve the sampling of G4 positions. The VolMap tool in VMD (4) was used to achieve an estimate of the sampling of G4 positions. Combined trajectories for each conformation containing every second frame of the raw output trajectories of both the restrained and non-restrained simulations for all rotational states were used in further analysis.

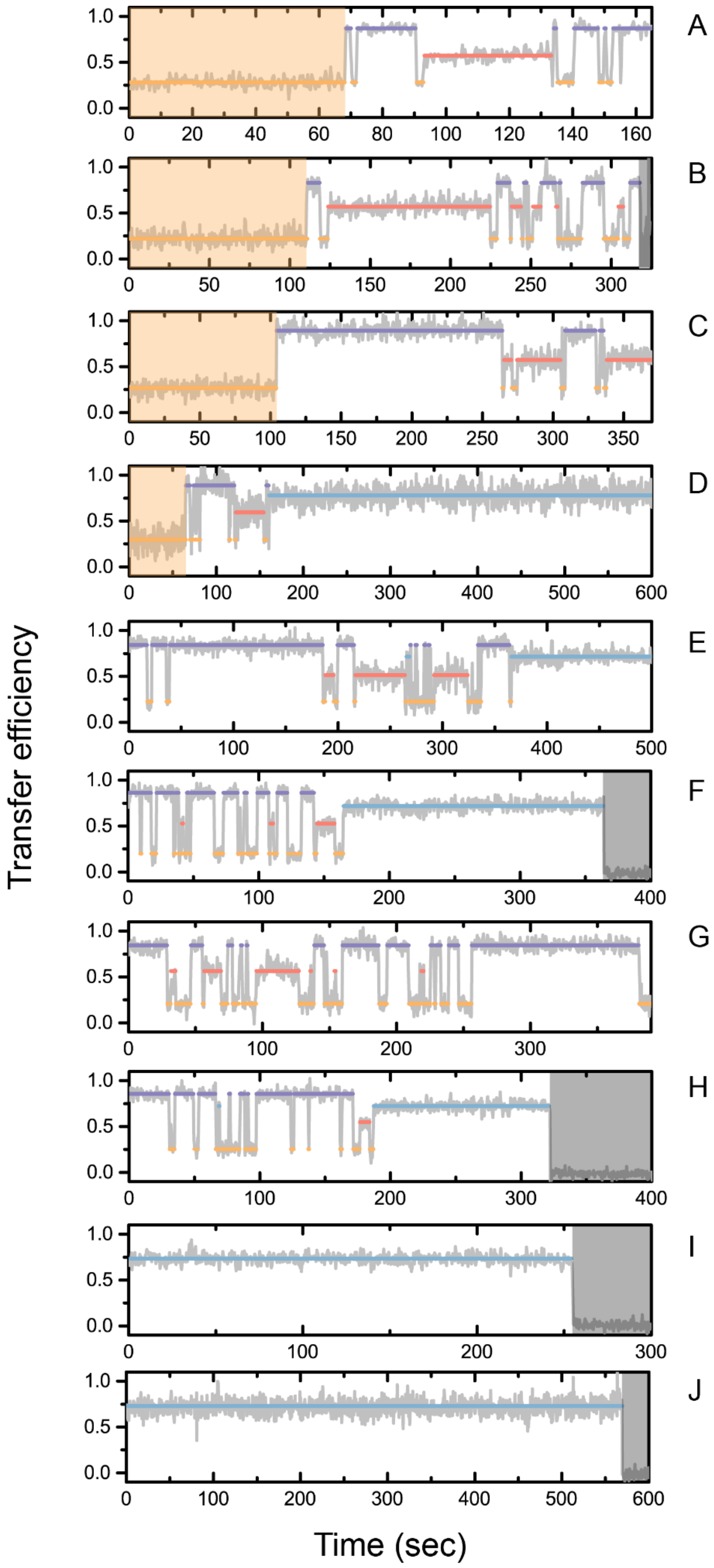

Figure S1. Representative single-molecule FRET trajectories revealing several FRET states observed upon folding of hTelo in the presence of 25 mM KCl. (A-D) FRET trajectories showing

real-time folding of G4 upon addition of KCl. The orange shaded area shows the time prior to KCl addition (within that time the sample was imaged in non-folding buffer). (E-H) FRET trajectories revealing fast conformational dynamics of G4 observed at later stages of the folding process. (I-J) Static FRET trajectory for G4 molecules stabilized in one state and showing no conformational dynamics. Colored solid lines indicate the FRET efficiency of these states as obtained from the HMM analysis. The grey shaded area shows the time after one or both fluorophores have photobleached. The color code is the same as in Fig. 1.

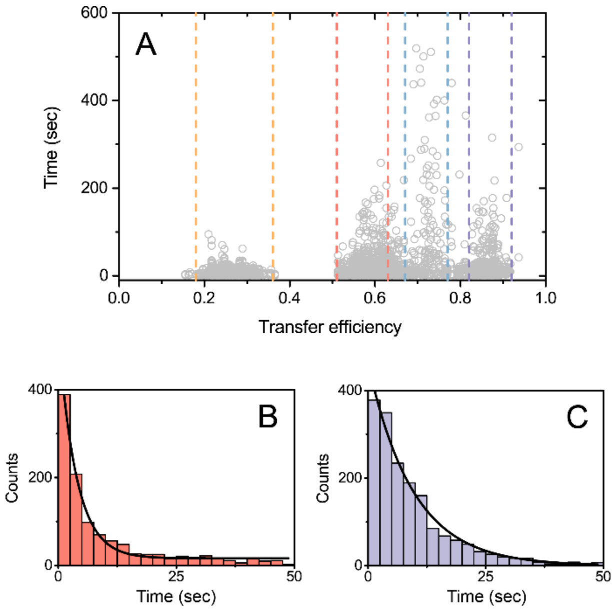

Figure S2. (A) Dwell times for human telomeric G4 in 25 mM KCl as a function of FRET efficiency, obtained from HMM analysis of single-molecule FRET trajectories. The colored dashed lines show the FRET ranges used for separating the dwell times corresponding to the four different FRET populations with mean transfer efficiencies of E≈0.30 (0.18-0.36), E≈0.57 (0.51-0.63), E≈0.73 (0.67-0.77) and E≈0.88 (0.82-0.92). (B, C) Dwell time histograms of the E≈0.57 and E≈0.88 states, respectively, fitted with a single-exponential functions (black line) with dwell times τ = 3.8 s and τ = 9.6 s, respectively. The dwell times for the E≈0.3 and E≈0.73 states (shown in in Figure 1 D, E) are τ = 5.3 s and τ_1 = 10 s, τ_2 = 100 s, respectively. Dwell time histograms for each E state (including those shown in Figure 1D, E) were built based on all dwell times (total residence time) for the corresponding states (including the dwell times cut by blinking, photobleaching or terminated by the end of the experiment). The color code is the same as in Fig. 1.

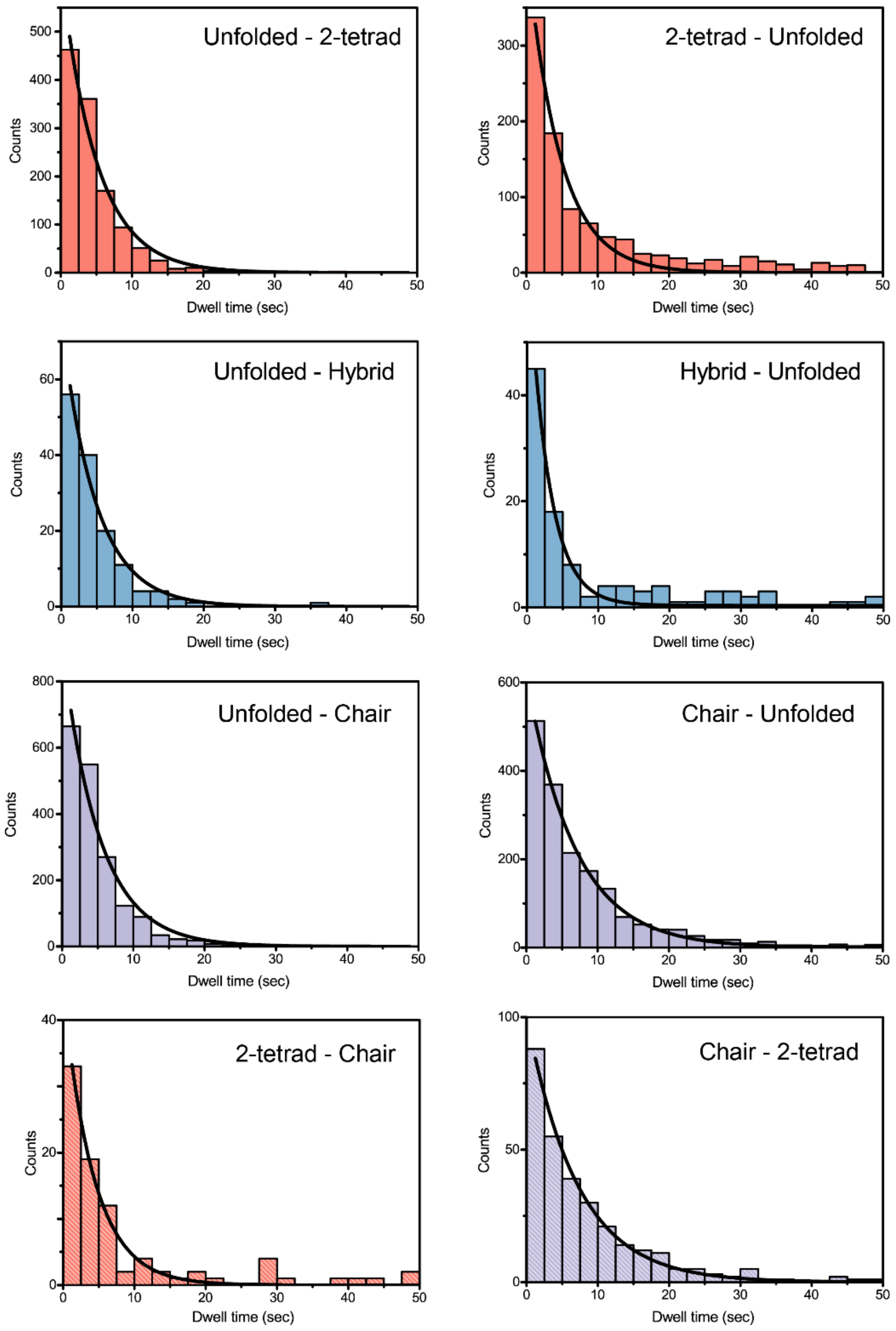

Figure S3. Dwell time histograms for transitions between two different G4 conformations. For example the dwell time histogram labelled with "Unfolded – 2-tetrad" includes the dwell times of molecules residing in an unfolded state prior transition to a 2-tetrad conformation, etc. The color code is the same as in Fig. 1. Because of similar FRET values of Hybrid 1 and Hybrid 2 conformations we could not distinguish the dwell times involving the transitions to and from these two conformations in this type of analysis and therefore they are labeled as "Hybrid". However, it should be noted that the dwell time histogram "Hybrid - Unfolded" will include mainly the data for the Hybrid 2 conformation, as the main long-lived Hybrid 1 conformation, usually appearing as a terminal state in FRET trajectories, will be excluded in this analysis.

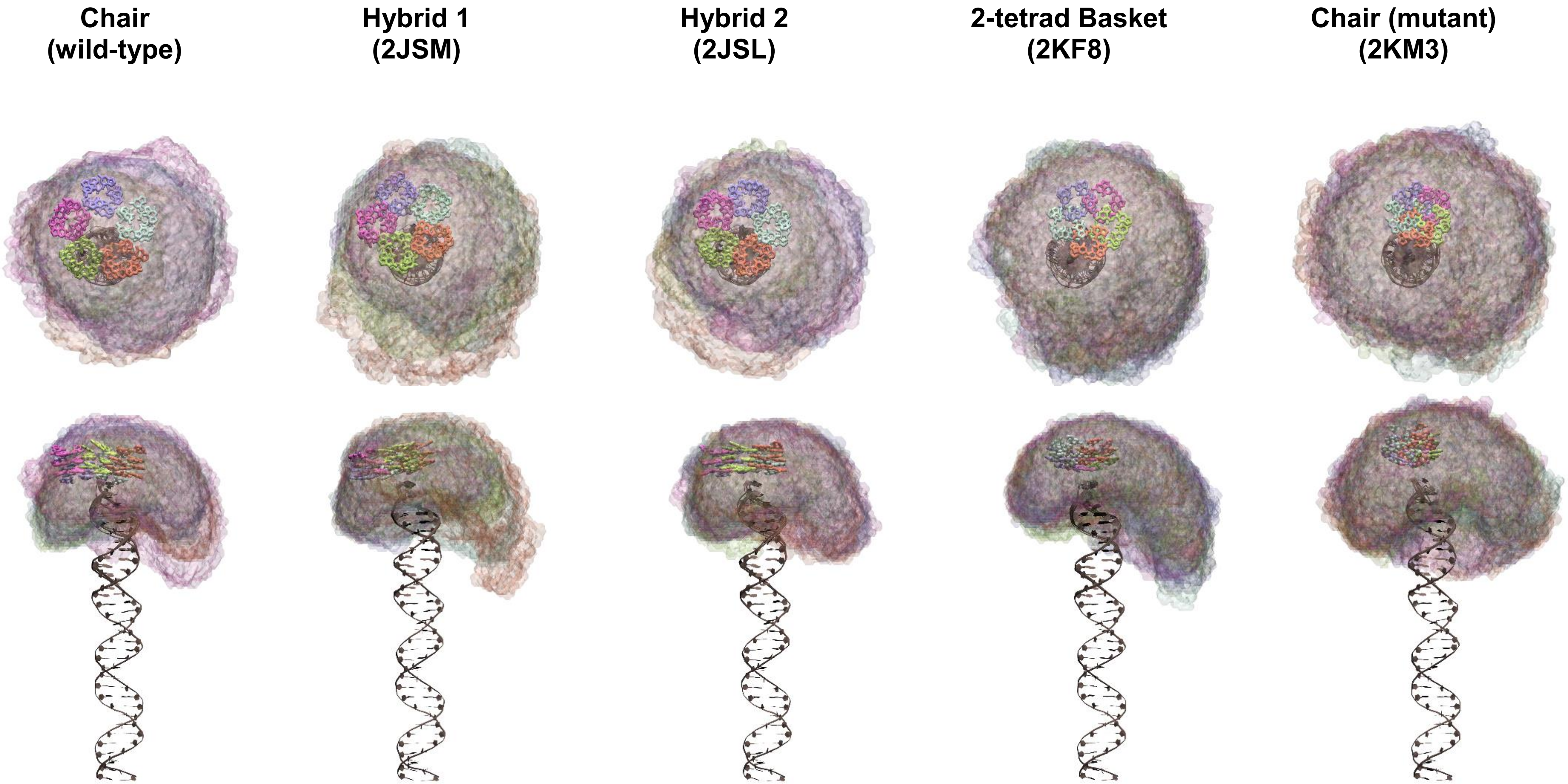

Figure S4. Rotational states and sampling of G4 positions. The five rotational starting positions of the G4s are shown in dark blue, light blue, orange, green, and magenta. The lightly colored spheres represent the space predominantly occupied by the G4 core nucleotides of the correspondingly colored starting conformation. The volumes predominantly occupied by the G-quartet nucleobases for the rotational states were found to overlap. This indicates that the simulations are independent of the starting conformations and that sampling is sufficient. The systems are shown from the top and from the side.

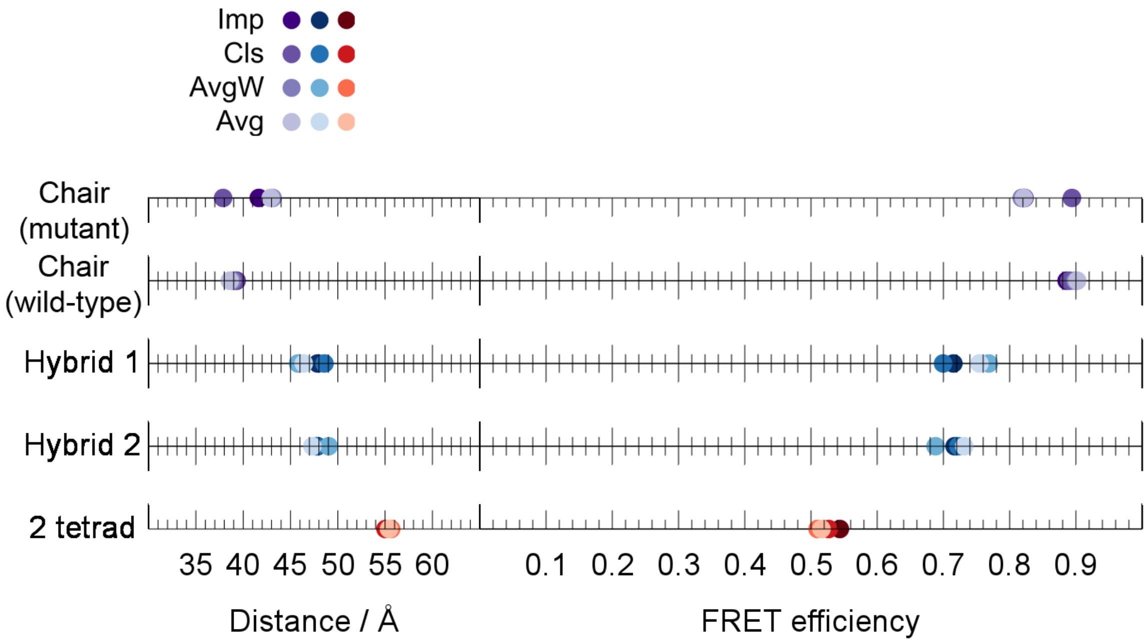

Figure S5. Distances between fluorophore mean positions and corresponding transfer efficiencies obtained from AV calculations. The transfer efficiencies were obtained using four approaches with different frames of the trajectories as input, as follows: 1) Conformations from every second frame of the simulations were included (very dark 'Imp' circles), 2) A cluster analysis was performed on G4 positions and all the clustered frames were included in the evaluation of FRET efficiencies (dark 'Cls' circles), 3) and 4) are based on average DNA conformations calculated from the frames of each of the ten clusters. When evaluating transfer efficiencies the ten efficiencies were either weighted by the number of frames in the clusters (light, 'AvgW') or averaged without weighting (very light, 'Avg'). The color code is consistent with the colors used for the different G4 conformations in the main text.

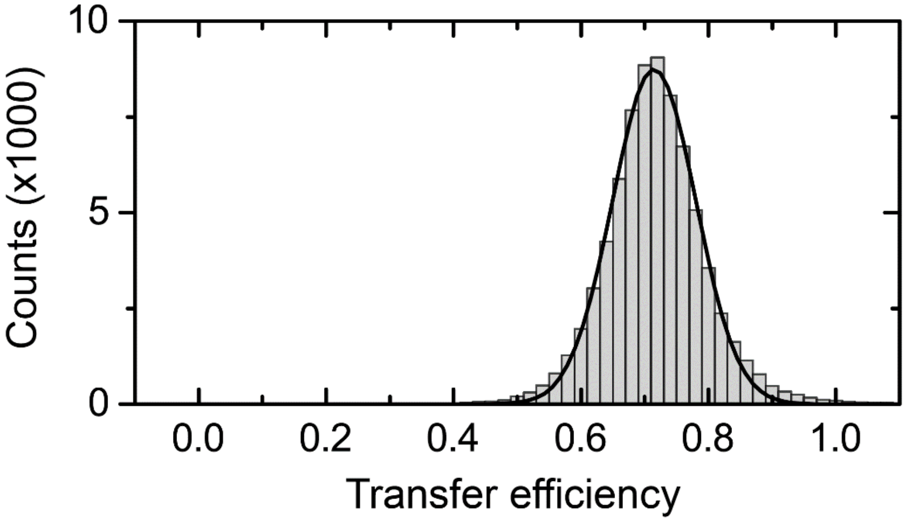

Figure S6. Single-molecule transfer efficiency histogram of the parallel G4 formed from c-Myc sequence in 25 mM KCl. The histogram was fitted to one Gaussian function (black line).

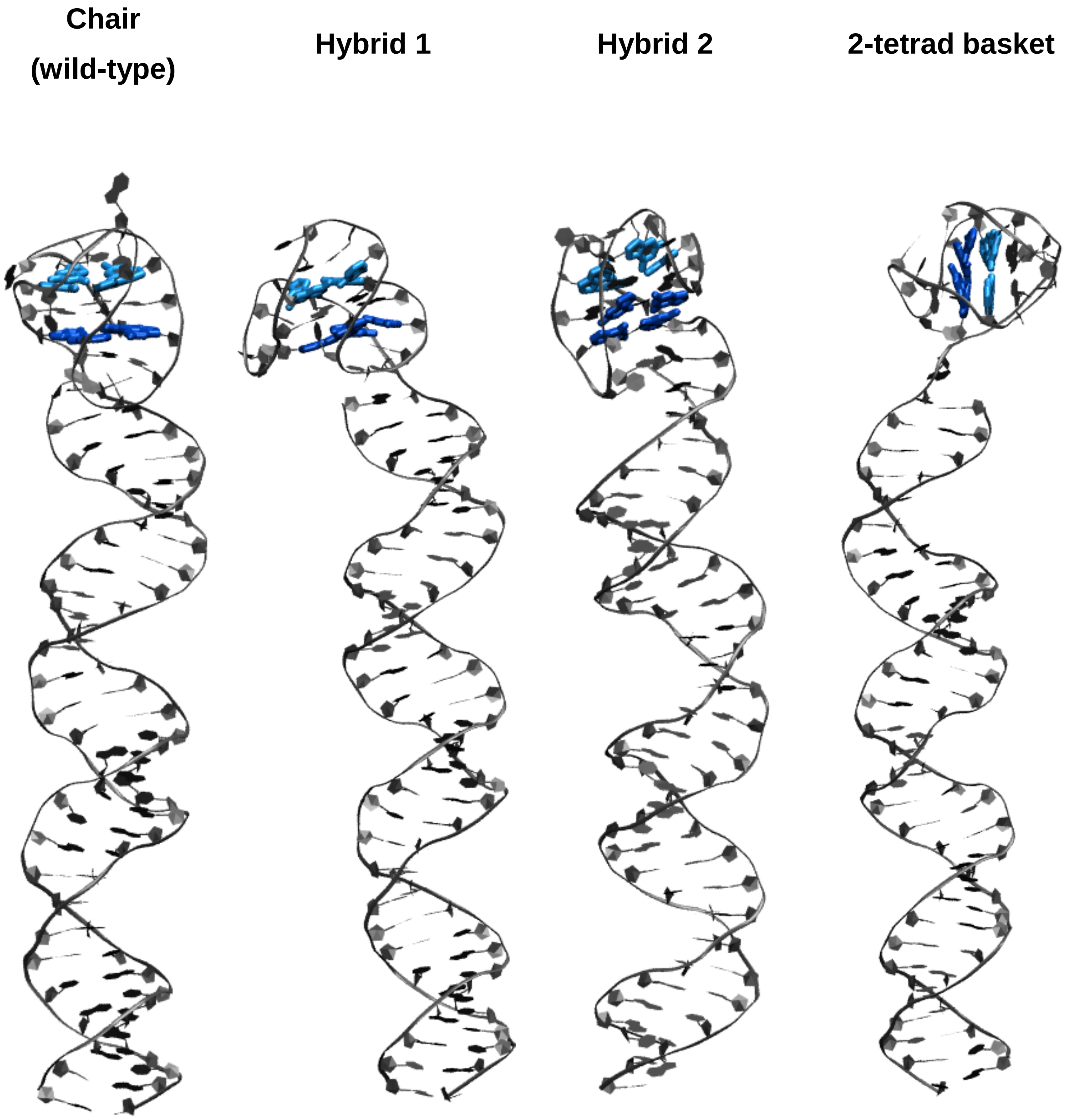

Figure S7. The representative structures of G4-duplex constructs for different G4 conformations as obtained from MD simulations. Each structure is an average of all structures from the dominant cluster 1 (Table S6). The first and last G4 tetrads are highlighted in dark and light blue, respectively. For the chair, hybrid 1 and hybrid 2 conformations the G4 tetrad plane is parallel to the plane of the last base pair of the duplex. For the 2-tetrad basket conformation the tetrads are positioned perpendicular to the last base pair plane.

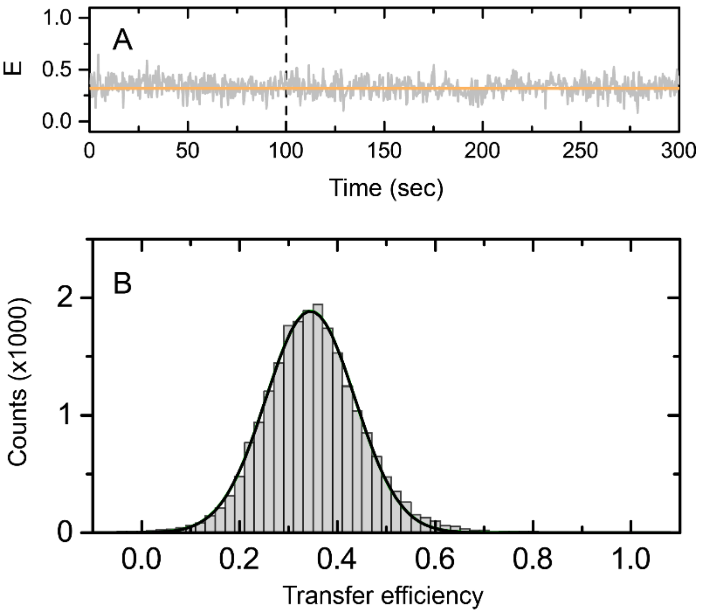

Figure S8. (A) Representative single-molecule FRET trajectory for the G-triplex forming sequence (see Table S1) upon injection of 25 mM KCl. Single molecules were initially imaged in 25 mM LiCl for 100 s, and then buffer with 25 mM KCl was manually injected to induce folding. The dashed line indicates the time of KCl injection. No change in transfer efficiency was observed upon buffer exchange. (B) Single-molecule transfer efficiency histogram for the G-triplex forming sequence in 25 mM KCl. The transfer efficiency distribution matches the one for the unfolded G4 overhang (Figure 1), indicating that even if G-triplex formation occurs under our experimental conditions this does not influence the transfer efficiency distribution, therefore excluding the possibility that the high E states may originate from such partially folded conformations.

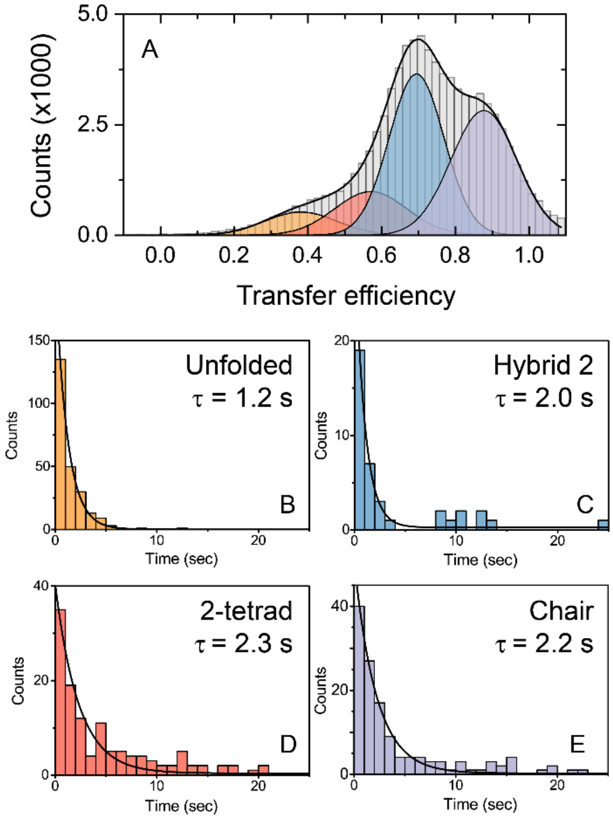

Figure S9. Single-molecule transfer efficiency histogram of the G4s formed from hTelo sequence in 100 mM KCl (A), acquired within 2 h after folding initiation. The histogram was fitted with a mix of four Gaussian functions (black lines). Dwell time histograms of the unfolded (B), hybrid 2 (C), 2-tetrad (D) and chair (E) G4 conformations formed from the hTelo sequence in the presence of 100 mM KCl. The colour code is the same as in Figure 1. The dwell time histograms were fitted by a single-exponential function (black lines) to extract the corresponding dwell times. In the presence of 100 mM KCl the G4 dynamics is significantly reduced resulting in only less than 20% of FRET trajectories showing dynamics between different states. The remaining 80% of trajectories are static within the experimental time window predominantly appearing at E≈0.7, corresponding to the most stable hybrid 1 state.

Table S1. List of DNA sequences used in this work

Name	Sequence
hTelo	(5’ - 3’) - GCA GGC GTG GCA CCG GTA ATA GGA TTA GGG TTA GGG TTA GGG TTA GGG TTA GGG(Cy3)
hTelo – m	(5’ - 3’) - GCA GGC GTG GCA CCG GTA ATA GGA TTA GGG **C**TA GGG **C**TA GGG **C**TA GGG **C**TA GGG(Cy3)
hTelo – ^{Br}G9,14	(5’ - 3’) - GCA GGC GTG GCA CCG GTA ATA GGA TTA GGG TTA GGG TTA G^{Br}**G**G TTA ^{Br}**G**GG TTA GGG(Cy3)
hTelo – ^{Br}G15	(5’ - 3’) - GCA GGC GTG GCA CCG GTA ATA GGA TTA GGG TTA GGG TTA GGG TTA G^{Br}**G**G TTA GGG(Cy3)
hTelo – I14	(5’ - 3’) - GCA GGC GTG GCA CCG GTA ATA GGA TTA GGG TTA GGG TTA GGG TTA **I**GG TTA GGG(Cy3) T
c-Myc	(5’ - 3’) - GCA GGC GTG GCA CCG GTA ATA GGA TTA GGG TTA GGG T GGG TA GGG T GGG(Cy3)
G-triplex	(5’ - 3’) - GCA GGC GTG GCA CCG GTA ATA GGA TTA GGG TTA GGG TTA GGG TTA GGG TTA TTT(Cy3)
Complementary duplex strand	(5’ - 3’) - AA CCC T(Cy5)AA TCC TAT TAC CGG TGC CAC GCC TGC(Biotin)

Table S2. The number of individual transitions observed in single-molecule FRET trajectories during G4 folding

Initial state \ Final state	Unfolded	2-tetrad	Hybrid 1&2	Chair
Unfolded		1198	140	1793
2-tetrad	1025		4	95
Hybrid 1&2	139	19		24
Chair	1776	308	41	

Table S3. The rate constants (s^{-1}) of individual transitions observed in single-molecule FRET trajectories during G4 folding

Initial state \ Final state	Unfolded	2-tetrad	Hybrid 1	Hybrid 2	Chair
Unfolded		0.20±0.01	0.21±0.01*	0.21±0.01*	0.19±0.01
2-tetrad	0.22±0.01		n.d.	n.d.	0.23±0.01
Hybrid 1	0.010±0.002**	n.d.		n.d.	n.d.
Hybrid 2	0.35±0.01	n.d.	n.d.		n.d.
Chair	0.150±0.002	0.140±0.002	n.d.	n.d.	

n.d. - the rate constants for the corresponding transitions were not determined due to the low number of transitions (Table S3) that was insufficient for building a dwell time histogram.
* The rate constants of folding of Hybrid 1 and Hybrid 2 are assumed to be the same.
** The rate constant of the unfolding of Hybrid 1 conformation is estimated from the double-exponential fit of the dwell time histogram of E=0.73 state (Figure 1E).

**Table S4.** Estimated unfolding free energy (ΔG_U) and the energy barriers of folding ($\Delta G_f^{\ddagger}$) and unfolding ($\Delta G_u^{\ddagger}$).			
	ΔG_U (kT)	$\Delta G_f^{\ddagger}$ (kT)	$\Delta G_u^{\ddagger}$ (kT)
Chair	0.24±0.06	15.48±0.07	15.71±0.01
2-tetrad	-0.10±0.02	15.42±0.06	15.33±0.04
Hybrid 2	-0.51±0.01	15.38±0.05	14.87±0.04
Hybrid 1	3.04±0.14	15.38±0.05	18.42±0.18

The free energy change for each transition was calculated using the following equation:

$$\Delta G_U = -k_B T ln\left(\frac{k_{unfold}}{k_{fold}}\right)$$

where the k_{fold} and k_{unfold} are the rate constants of the folding and unfolding, respectively, $k_B=1.381 \cdot 10^{-23}$ J K^{-1} is the Boltzman's constant and T=295 K is the reference temperature.

The height of the folding and unfolding energy barriers were estimated through the following equations:

$$\Delta G_f^{\ddagger} = -k_B T ln\left(\frac{k_{fold}}{k_w}\right) \quad \text{and} \quad \Delta G_u^{\ddagger} = -k_B T ln\left(\frac{k_{unfold}}{k_w}\right)$$

where k_w is the frequency factor that is estimated to be ~10^6 s^{-1} (5,6).

**Table S5.** Frame distribution of cluster analyses					
Cluster	Chair (wild-type)	Hybrid 1 (2JSM)	Hybrid 2 (2JSL)	2-tetrad (2KF8)	Chair (mutant)* (2KM3)
1	31%	40%	43%	9%	24%
2	8%	9%	9%	6%	7%
3	7%	8%	8%	6%	5%
4	5%	5%	4%	5%	3%
5	3%	4%	4%	4%	3%
6	3%	3%	2%	4%	2%
7	3%	2%	2%	4%	2%
8	2%	2%	1%	4%	2%
9	2%	2%	1%	3%	2%
10	2%	1%	1%	3%	2%

*2 µs simulations with 10,000 frames.
All other simulations were 1.5 µs simulations with 7,500 frames.

**Table S6.** Dye parameters						
Dye	Radius 1	Radius 2	Radius 3	Linker length	Linker width	Attachment*
Cy3	8.2 Å	3.0 Å	2.2 Å	20 Å	4.5 Å	G4
Cy5	9.5 Å	3.0 Å	2.2 Å	22 Å	4.5 Å	Duplex

* The O3´ of the last nucleotide in the G4s was used as attachment point for Cy3 and the methyl group carbon atom of T7 in the complementary strand was used as attachment point for Cy5. Before starting the AV calculations, hydrogen atoms attached to this O3’ and this C were deleted.

REFERENCES

1. Maestro. (2012). 9.3 ed. Schrödinger LLC, New York, NY.
2. Mashimo, T., Yagi, H., Sannohe, Y., Rajendran, A. and Sugiyama, H. (2010) Folding pathways of human telomeric type-1 and type-2 G-quadruplex structures. *J. Am. Chem. Soc.*, **132**, 14910-14918.
3. Konig, S.L., Huppert, J.L., Sigel, R.K. and Evans, A.C. (2013) Distance-dependent duplex DNA destabilization proximal to G-quadruplex/i-motif sequences. *Nucleic Acids Res.*, **41**, 7453-7461.
4. Humphrey, W., Dalke, A. and Schulten, K. (1996) VMD: visual molecular dynamics. *J. Mol. Graph.*, **14**, 33-38.
5. Woodside, M.T., Behnke-Parks, W.M., Larizadeh, K., Travers, K., Herschlag, D. and Block, S.M. (2006) Nanomechanical measurements of the sequence-dependent folding landscapes of single nucleic acid hairpins. *Proc. Natl. Acad. Sci. USA*, **103**, 6190-6195.
6. Thirumalai, D. and Hyeon, C. (2009) In Walter, N. G., Woodson, S. A. and Batey, R. T. (eds.), *Non-Protein Coding RNAs*. Springer Berlin Heidelberg, Berlin, Heidelberg, pp. 27-47.
